# Repair of aortic coarctation in neonates less than two kilograms

**DOI:** 10.1093/icvts/ivae185

**Published:** 2024-12-02

**Authors:** Qiang Chen, Thomas Fleming, Massimo Caputo, Serban Stoica, Andrew Tometzki, Andrew Parry

**Affiliations:** Department of Pediatric Cardiothoracic Surgery, Hong Kong Children’s Hospital, Hong Kong, China; Department of Cardiac Surgery, Bristol Royal Hospital for Children, University of Bristol, Bristol, UK; Department of Cardiac Surgery, Bristol Royal Hospital for Children, University of Bristol, Bristol, UK; Department of Cardiac Surgery, Bristol Royal Hospital for Children, University of Bristol, Bristol, UK; Department of Cardiac Surgery, Bristol Royal Hospital for Children, University of Bristol, Bristol, UK; Department of Cardiology, Bristol Royal Hospital for Children, University of Bristol, Bristol, UK; Department of Cardiac Surgery, Bristol Royal Hospital for Children, University of Bristol, Bristol, UK

**Keywords:** Congenital Heart Disease, Coarctation of aorta, Low birth weight, Neonates

## Abstract

**OBJECTIVES:**

A significant number of low-birth-weight neonates are born with aortic coarctation. Previous studies of early operation on these patients have shown a high hospital mortality and recurrence at 1 year. We reviewed our data to ascertain whether modern approaches allow better outcomes for these children.

**METHODS:**

Fourteen patients weighing <2 kg with isolated coarctation between January 2005 and December 2015 were studied by retrospective chart review to ensure >5 years follow-up. All patients underwent extended end-to-side surgical repair. In-hospital and medium-term follow-up data were collected. Data are expressed as median (range).

**RESULTS:**

Weight at the time of surgery was 1.8 (1.5–1.9) kg. There were no deaths, in-hospital or during follow-up. In-hospital stay was 11 (4–47) days. At follow-up of 141 (80–207) months echocardiographic velocity across the repair was 1.6 (0.9–3.8) m/s. Two patients required balloon dilatations for recoarctation including 1 with William’s syndrome who required balloon coarctoplasty followed by stenting. This patient had grossly abnormal vessels at the time of initial surgery with aortic wall thickness >3 mm. There were no central neurological complications. Other complications included vocal cord dysfunction in 1, development of chylothorax requiring prolonged chest drainage in 2, pneumothorax following chest drain removal in 1 and wound dehiscence in 1 patient.

**CONCLUSIONS:**

Neonates below 2 kg can undergo coarctation repair safely with low incidence of recurrence. Waiting for growth in this cohort of patients may not therefore be justified.

## Graphical Abstract

**Figure:**
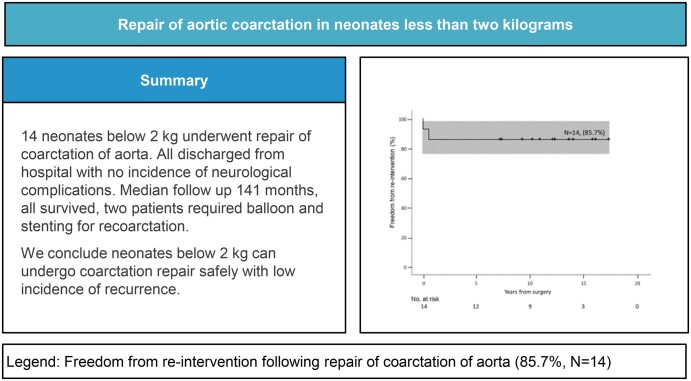

## INTRODUCTION

The conventional treatment of aortic coarctation in low birth weight and premature neonates is to pursue initial conservative management with Prostaglandin infusion followed by surgical repair when the child is of ‘adequate’ size. However, waiting for the child to grow before intervening rarely permits significant useful weight gain and puts the child at risk of interim morbidity. Previous studies of early operation on these patients, however, have shown a high mortality (up to 24%) [1] and recurrence (up to 44%) [2] at 1 year.

In our institution, we pursued an aggressive approach of early surgical intervention when the child presented. In this study, we reviewed our data to ascertain whether this approach, along with modern surgical and postoperative techniques, allows better outcomes for these children.

## METHODS

Fourteen premature neonates with birth weight <2 kg underwent extended end-to-side surgical repair of isolated aortic coarctation between January 2005 and December 2015. Patients with proximal arch hypoplasia required median sternotomy approach and cardiopulmonary bypass, and those with other lesions required concomitant or staged open heart operations were excluded from the study. The cut-off date was selected to ensure at least 5 years of follow-up. The median age at the time of surgery was 11 (2–28) days and weight 1.8 (1.5–1.9) kg. The median gestational age was 32 (30–34) weeks. The diagnosis was confirmed in all patients by echocardiography. Only patients with classical isolated coarctation of the thoracic aorta were included in this study to exclude confounding factors, although all patients also had a patent ductus arteriosus. Associated co-mobilities include tracheoesophageal fistula in 1, William Syndrome in 1, infant respiratory distress syndrome in 1, suspected necrotizing enterocolitis in 2 and Chromosome 4Q/7Q/10Q deletion in 1. Prostaglandin infusion was used in 12 patients prior to surgery. The associated cardiac lesions include bicuspid aortic valve in 2, small ventricular septal defect in 1, mild aortic stenosis in 1, mild mitral stenosis in 1 and bilateral superior vena cava in 1.

### Ethics committee approval

This was a retrospective chart review, and Ethics Committee approval was waived.

### Patient and public involvement

There were no patient and public involvement in this study.

### Surgical technique

A standard surgical technique was used [3]. Briefly, after appropriate anaesthetic preparation, monitoring was performed using a right arm arterial line and cerebral near-infrared spectroscopy. Through a left thoracotomy incision, entering the chest through the 3rd intercostal space, the preparatory dissection was performed during which time the patient was allowed to cool so that at the time of aortic clamping, core temperature was 34–35°C.

An extensive dissection of the aorta was performed dissecting from distal ascending aorta to mid-descending thoracic aorta along with the intrathoracic portions of the head and neck arteries and the patent ductus arteriosus. The patent ductus arteriosus was ligated with a purse-string 5–0 Polypropylene suture. A side-biting clamp was applied to the aortic arch, taking it as far anteriorly as necessary to get proximal to any arch narrowing whilst maintaining adequate flow to the brain via the innominate artery as assessed by the arterial line trace and the near-infrared spectroscopy monitor. Frequently, this clamp encroached on the origin of the innominate artery on which occasions it was confirmed that manipulation of the clamp, as would be necessary later to perform the anastomosis, still allowed adequate cerebral perfusion. After applying a cross-clamp to the descending thoracic aorta, the coarctation segment was aggressively excised, trimming the descending thoracic aorta back as far as necessary to remove all ductal tissue; this can be confidently performed in small neonates as the arteries are enormously elastic. The opening in the distal arch was enlarged by incising the under surface of the aortic arch, taking this incision as far anteriorly as necessary to get proximal to any narrowing. Aortic continuity was then re-established with an (extended) end-to-side anastomosis performed using a running 7–0 Polypropylene suture and the excluded aortic segment was deaired prior to tying down the suture line. The rest of the operation was performed in a routine fashion.

Post-operatively, the child was cared for by a Neonatal Intensive Care Unit (NICU) nurse, initially on the Paediatric Intensive Care Unit (PICU). The child was transferred back to NICU as soon as it was safe, usually on the day following surgery, with further input from the surgical team.

### Data analysis

Data are expressed as median (range). In the case of the survival and freedom from reoperation curves, the data were processed after the Kaplan–Meier method using the R Core Team (2013) statistical package (R Foundation for Statistical Computing, Vienna, Austria; http://www.R-project.org/).

## RESULTS

Aortic cross-clamp time was 16 (13–20) min. There were no deaths, and no patient developed postoperative central neurological deficit.

One patient had vocal cord dysfunction during the early postoperative period. Other complications included development of chylothorax requiring prolonged chest drainage in 2, pneumothorax following chest drain removal in 1 and wound dehiscence in 1 patient.

The median postoperative ventilation time and length of stay in the intensive care unit and on the ward were 3 (1–7), 4 (2–9) and 11 (4–47) days, respectively.

**Figure 1:**
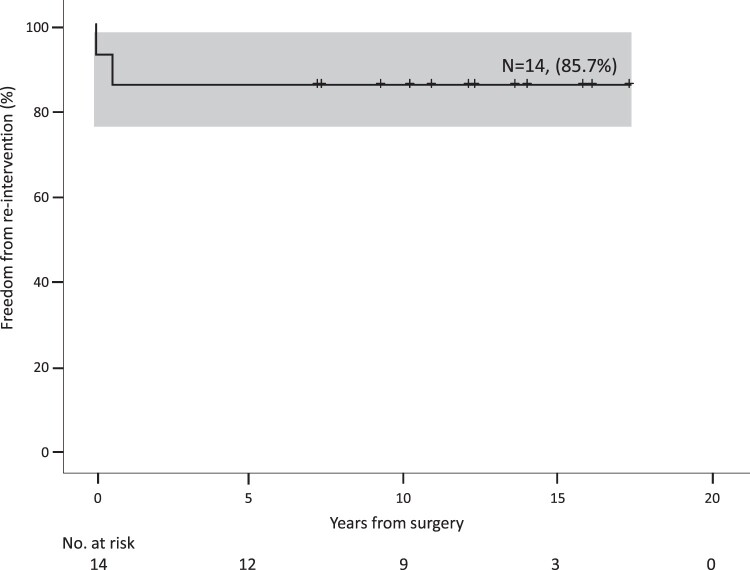
Freedom from reintervention for recoarctation in neonates <2 kg underwent repair of coarctation of aorta.

**Figure 2:**
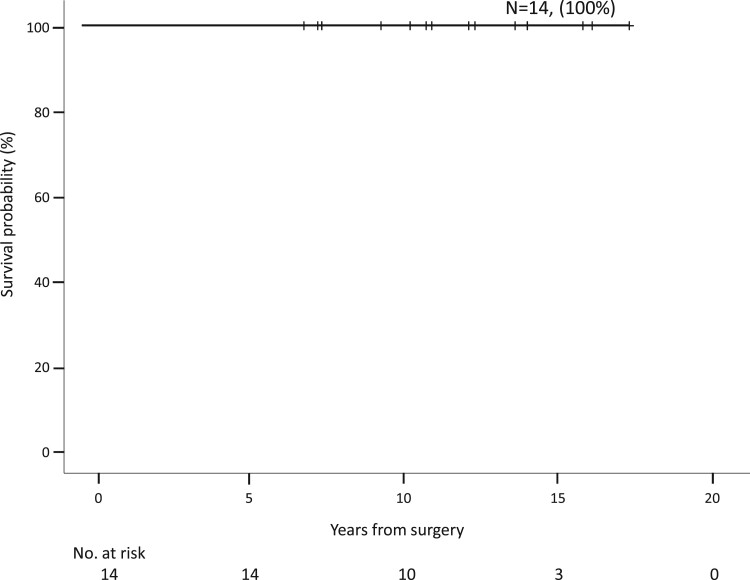
Kaplan–Meier survival for neonates <2 kg underwent repair of coarctation of aorta.

**Figure 3:**
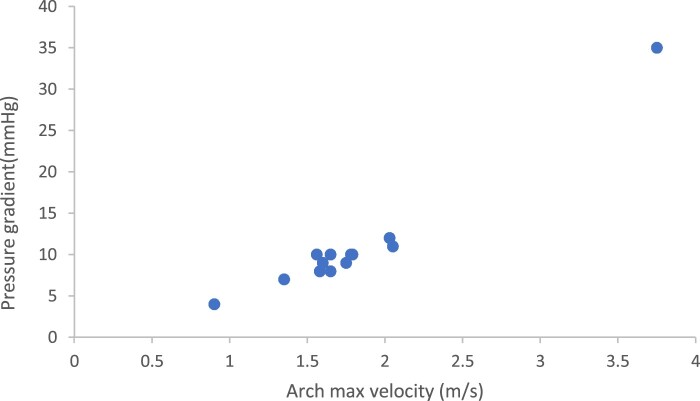
Scatter graph of aortic arch velocity (m/s) and clinical pressure gradient (mmHg) following repair of aortic coarctation in neonates <2 kg.

Follow-up was complete in all patients at 141 months (80–207). No child exhibited clinical evidence of neurological deficits. Freedom from reintervention for recoarctation at 5th year was 85.7% with 95% CI from 79.4% to 100% (Fig. 1). All patients survived, and the survival was 100% at 5 years (Fig. 2). At follow-up, the mean echocardiographic velocity across the repair was 1.6 (0.9–3.8) m/s. The median clinical pressure gradient was 9.5 mmHg (95% CI 4–35). The arch velocity and clinical pressure gradient are shown in Fig. 3. None of the patients required antihypertension medications. One patient required balloon coarctoplasty 3 months and 35 months after surgery. Another patient associated with William’s syndrome required balloon coarctoplasty followed by stenting for recoarctation 4 months after the operation; this patient had grossly abnormal vessels at the time of initial operation with aortic wall thickness >3 mm.

## DISCUSSION

The optimal management of low-birth-weight neonates with critical aortic coarctation remains controversial. Whilst some report that low weight does not affect outcomes after coarctation repair [4], others reported unfavourable early survival and high reintervention rate with very low birth weight a multivariable predictor for recoarctation [1, 2]. Many surgeons may choose to delay the repair in order to achieve growth. However, this approach is not without risk. It has been found that there is an increased risk of intraventricular haemorrhage associated with the presence of coarctation in low-birth-weight infants with an odds ratio of 2.9, though whether earlier intervention reduces this risk is as yet unproven [5]. Although Prostaglandin infusion is acutely effective [6] when prolonged, it is associated with a number of side effects including apnoea, convulsions, oedema and Gastrointentestinal intolerance [7]. The surgical impact of the oedema is that it gives a false sense that the child’s weight is increasing, whereas in fact all that is happening is that the operation is becoming more complex. Up to 43% of patients waiting for surgery on long-term Prostaglandin suffer 1 such event though the majority can be managed with minor changes in management [8]. Although this is an old study, the overall mortality during the period of waiting was 31% with neonates with left-sided obstructive lesions being at twice the risk of those with cyanotic heart lesions.

Concern with this approach and an increased confidence in operating on small neonates gained through team experience encouraged us to undertake surgical correction of coarctation in low-birth-weight neonates rather than the wait and grow approach. With sufficient experience, we, like others, have been able to use this approach with a 0% early and late mortality (published mortality range 0–24%) [1, 4, 9, 10]. This reflects the experience of the whole team, pre-, peri- and postoperatively. Personal experience of patient outcomes being more dependent on complex neonatal management issues rather than the performance of the surgical team led us to have the patient cared for by NICU nurses, on PICU for the 1st post-surgical night, with transfer back to NICU as soon as safe, usually the day following surgery. This enabled us to have very few complications.

Inevitably, operating on small neonates raises the concern of recurrence due to the small physical size of the patient and vessel. Reports vary widely on the incidence of recoarctation with rates ranging from 15% to 44% [2, 11]. In our series, 2 patients (14%) required further intervention following surgery, and 1 of them had structurally grossly abnormal arterial walls, later being diagnosed to have William’s syndrome.

We consider that the surgical approach is crucial in this regard and highlight 3 factors:

- complete resection of all coarctation/ductal tissue (this is possible in the neonate as the tissues are extremely extensile)
- extensive dissection of aortic arch and descending thoracic aorta to minimize tension on the anastomosis
- incising the concavity of the aortic arch and outside of the descending thoracic aorta to spatulate and thereby enlarge the anastomosis

Potential alternatives to surgery exist, specifically balloon aortoplasty and stent placement. Whilst neither is strongly advocated as definitive treatment of coarctation in neonates, they might enable temporization of the obstruction for long enough for the child to grow adequately to allow definitive surgery to be performed.

There have been a small number of reports of successful palliative aortoplasty in extremely low-birth-weight infants using a variety of vascular routes [12–14]. Though often initially successful, early recoarctation is common and loss of vascular access often precludes further dilatations [14]. Repeat dilatation has been performed with success, but other reinterventions have precipitated more extensive surgical reconstructions with little time, and therefore growth, between them. In 1 series of 6 low-birth-weight neonates under 2.5 kg while balloon dilatation was successful in all, only 3 required no further intervention at 42 months; the other 3 developed restenosis within 3 months of the intervention and proceeded to a 2nd dilatation [14]. Two of these infants had a restenosis and required surgery within 2 months of the redilatation (1 of them developed recoarctation following surgery and required further balloon dilatation). Similarly, Sutton *et al.* reported 4 cases, only 1 of whom was discharged after successful balloon coarctoplasty [15]. One died in hospital, and the other 2 required multiple reinterventions including 1 required early surgical reintervention. Experience with stenting of the stenosis has been mixed. In a series of 5 patients weighing under 1.5 kg, 1 patient developed severe restenosis with aneurysm formation, which was managed using covered stents [5]. Surgery was later performed following stenting, and during follow-up (0.1–5 years), no reinterventions have been required. However, in all patients, the femoral artery (site of vascular access) was occluded. This complication was avoided by Cools *et al.* [16] who successfully stented a coarctation through a median sternotomy, allowing stent removal and arch repair 5 months later. In an attempt to enable prolonged palliation Sallmon *et al.* reported using bioresorbable stents, but with poor outcomes. Patient developed multiple early severe restenosis of the sirolimus-eluting stent, requiring early corrective surgery [17].

### Limitations and conclusions

None of the patients in this study were <1 kg, and whether our results are applicable in this cohort remains unproven. The single institution nature of this study limits the generalizability of the results.

A retrospective study without a comparative group. Due to small number of cases, we did not compare this tightly defined cohort of patients with those hypoplastic arch required median sternotomy approach and those required concomitant or staged open heart operations. Furthermore, the neurological assessments were not formalized using a scoring system. However, our consecutive operating results observed that neonates below 2 kg can undergo coarctation repair safely with low incidence of recurrence requiring reintervention in the short and median terms. Our result supports the hypothesis that early repair, along with modern surgical and postoperative care, could achieve optimal outcomes in neonates below 2 kg who underwent repair of isolated aortic coarctation. The long-term benefits of early repair necessitate a prospective randomized controlled study.

## Data Availability

The data underlying this article will be shared on reasonable request.

## References

[1] Karamlou T , Bernasconi A, Jaeggi E, Alhabshan F, Williams WG, Van Arsdell GS et al Factors associated with arch reintervention and growth of the aortic arch after coarctation repair in neonates weighing less than 2.5 kg. J Thorac Cardiovasc Surg 2009;137:1163–7. 19379984 10.1016/j.jtcvs.2008.07.065

[2] Bacha EA , Almodovar M, Wessel DL, Zurakowski D, Mayer JE Jr, Jonas RA et al Surgery for coarctation of the aorta in infants weighing less than 2 kg. Ann Thorac Surg 2001;71:1260–4. 11308171 10.1016/s0003-4975(00)02664-3

[3] Jonas RA. Coarctation of the aorta, Chapter 12. In: Comprehensive Surgical Management of Congenital Heart Disease, 1st edn. London: Hodder Arnold Publication, 2004, 214.

[4] Sudarshan CD , Cochrane AD, Jun ZH, Soto R, Brizard CP. Repair of coarctation of the aorta in infants weighing less than 2 kilograms. Ann Thorac Surg 2006;82:158–63. 16798207 10.1016/j.athoracsur.2006.03.007

[5] Stegeman R , Breur JMPJ, Heuser J, Jansen NJG, de Vries WB, Vijlbrief DC et al Primary coronary stent implantation is a feasible bridging therapy to surgery in very low birth weight infants with critical aortic coarctation. Int J Cardiol 2018;261:62–5. 29550016 10.1016/j.ijcard.2018.03.009

[6] Seckeler MD , White SC, Fox KA. Increased risk of intraventricular hemorrhage in low birth weight infants with aortic coarctation. J Matern Fetal Neonatal Med 2018;19:1–3. 10.1080/14767058.2018.1517319 30149745

[7] Freed MD , Heymann MA, Lewis AB, Roehl SL, Kensey RC. Prostaglandin E1 infants with ductus arteriosus-dependent congenital heart disease. Circulation 1981;64:899–905. 7285305 10.1161/01.cir.64.5.899

[8] Cucerea M , Simon M, Moldovan E, Ungureanu M, Marian R, Suciu L. Congenital heart disease requiring maintenance of ductus arteriosus in critically ill newborns admitted at a tertiary neonatal intensive care unit. J Crit Care Med (Targu Mures) 2016;2:185–91. 29967858 10.1515/jccm-2016-0031 PMC5953253

[9] Lewis AB , Freed MD, Heymann MA, Roehl SL, Kensey RC. Side effects of therapy with Prostaglandin E1 in infants with critical congenital heart disease. Circulation 1981;64:893–8. 7285304 10.1161/01.cir.64.5.893

[10] Curzon CL , Milford-Beland S, Li JS, O'Brien SM, Jacobs JP, Jacobs ML et al Cardiac surgery in infants with low birth weight is associated with increased mortality: analysis of the Society of Thoracic Surgeons Congenital Heart Database. J Thorac Cardiovasc Surg 2008;135:546–51. 18329467 10.1016/j.jtcvs.2007.09.068

[11] Bové T , François K, De Groote K, Suys B, De Wolf D, Verhaaren H et al Outcome analysis of major cardiac operations in low weight neonates. Ann Thorac Surg 2004;78:181–7. 15223425 10.1016/j.athoracsur.2003.12.066

[12] Burch PT , Cowley CG, Holubkov R, Null D, Lambert LM, Kouretas PC et al Coarctation repair in neonates and young infants: is small size or low weight still a risk factor? J Thorac Cardiovasc Surg 2009;138:547–52. 19698833 10.1016/j.jtcvs.2009.04.046

[13] Prada F , Carretero J, Mortera C, Velasco D. Balloon angioplasty in a 1200-gram premature infant with critical aortic coarctation. Rev Esp Cardiol 2010;63:740–50. 10.1016/s1885-5857(10)70151-1 20515634

[14] Rothman A , Galindo A, Evans WN, Collazos JC, Restrepo H. Effectiveness and safety of balloon dilation of native aortic coarctation in premature neonates weighing < or = 2,500 grams. Am J Cardiol 2010;105:1176–80. 20381673 10.1016/j.amjcard.2009.12.023

[15] Sutton N , Lock JE, Geggel RL. Cardiac catheterization in infants weighing less than 1,500 grams. Catheter Cardiovasc Interv 2006;68:948–56. 17086522 10.1002/ccd.20905

[16] Cools B , Meyns B, Gewillig M. Hybrid stenting of aortic coarctation in very low birth weight premature infant. Catheter Cardiovasc Interv 2013;81:E195–8. 22431483 10.1002/ccd.24420

[17] Sallmon H , Berger F, Cho MY, Opgen-Rhein B. First use and limitations of Magmaris bioresorbable stenting in a low birth weight infant with native aortic coarctation. Catheter Cardiovasc Interv 2019;93:1340–3. 31001884 10.1002/ccd.28300

