# Corrigendum to: Repair of aortic coarctation in neonates less than two kilograms

**DOI:** 10.1093/icvts/ivag220

**Published:** 2026-08-14

This is a corrigendum to: Qiang Chen, Thomas Fleming, Massimo Caputo, Serban Stoica, Andrew Tometzki, Andrew Parry, Repair of aortic coarctation in neonates less than two kilograms, *Interdisciplinary CardioVascular and Thoracic Surgery*, Volume 39, Issue 6, December 2024, ivae185, https://doi.org/10.1093/icvts/ivae185

In the originally published version of this manuscript, a source of grant support was omitted.

The following statement should have been included in the ‘Funding’ section:

“This work was supported by a grant from the British Heart Foundation; grant number CH/17/1/32804”.

This error has been corrected only in this correction notice to preserve the version of record.

